# Supplementary material for: TSSpredator-Web: A web-application for transcription start site prediction and exploration
Source: PLoS One. 2026 Mar 13;21(3):e0326299. doi: 10.1371/journal.pone.0326299 (PMC12987492; doi:10.1371/journal.pone.0326299)
Supplement: Supplementary Material — (PDF) [file pone.0326299.s005.pdf]

Supplementary material for TSSpredator-Web: A Web-Application for Transcription Start Site Prediction and Exploration

Mathias Witte Paz, Alexander Herbig & Kay Nieselt

A The TSSpredator algorithm

Algorithm S1 Library normalization of TSSpredator

Input: $r \in N$ with N is the number of coverage profile pairs (samples)
C^r_{non}: Normalized non-enriched coverage profiles r
C^r_{enr}: Normalized enriched coverage profiles r
PL: Percentile for library normalization, default $PL = 90$
PE: Percentile for enrichment normalization, default $PE = 50$
Output: Library normalized coverage profiles e^r_{non} and e^r_{enr}

$Q \leftarrow \emptyset$
$EF \leftarrow \emptyset$
for all $r \in N$ **do**
  $Q^r \leftarrow$ getPercentile(C^r_{enr}, PL)
  $Q \leftarrow \{Q^i, Q\}$
  for $s \in \{+, -\}$ **do**
    $N^{r,s}_{enr} \leftarrow C^{r,s}_{enr}/Q^i$
    $N^{r,s}_{non} \leftarrow C^{r,s}_{non}/Q^i$
  end for
end for
$Q_{min} \leftarrow \min(Q)$
for all $s \in \{+, -\}$ and $i \in N$ **do**
  $N^{*r,s}_{enr} \leftarrow N^{r,s}_{enr} * Q_{min}$
  $N^{*r,s}_{non} \leftarrow N^{r,s}_{enr} * Q_{min}$
  $\mathcal{T} \leftarrow$ predictTSS$(N^{*r,s}_{enr}, N^{*r,s}_{non})$
  $F_r \leftarrow$ getEnrichmentFactors$(\mathcal{T})$
  $EF_r \leftarrow$ getPercentile(F_r, PE)
  $EF \leftarrow \{EF_r, EF\}$
end for
$EF_{max} \leftarrow \max(EF)$
for all $s \in \{+, -\}$ and $r \in N$ **do**
  $e^{r,s}_{non} \leftarrow N^{*r,s}_{non} * \frac{EF_i}{EF_{max}}$
  $e^{r,s}_{enr} \leftarrow N^{*r,s}_{enr}$
end for

Algorithm S2 Helper functions for TSS prediction

function DETECTTSSPERREPLICATE($e^r_{enr}, Q_{min}, T_h, T_f, W$)
  $T_h^{\text{abs}} \leftarrow T_h \cdot Q_{min}$, $\mathcal{D} \leftarrow \emptyset$
  for $i = 2$ to L **do**
    $\Delta h \leftarrow e^r_{enr}(i) - e^r_{enr}(i-1)$, $F \leftarrow e^r_{enr}(i)/e^r_{enr}(i-1)$
    if $\Delta h \geq T_h^{\text{abs}}$ **and** $F \geq T_f$ **then**
      $\mathcal{D} \leftarrow \{\mathcal{D}, i\}$
    end if
  end for
  return COLLAPSECLOSEPOSITIONS($\mathcal{C}$, W)
end function

function GETENRICHMENTFACTORACROSSREPLICATES($\mathcal{T}_k^{detected}(j), e^r_{k,non}, e^r_{k,enr}$)
  $EF_{max} \leftarrow 0$
  for all replicates r of condition k **do**
    $EF \leftarrow e^r_{enr}(j)/e^r_{non}(j)$
    $\max(EF_{max}, EF)$
  end for
  return EF_{max}
end function

Algorithm S3 TSS prediction with TSSPREDATOR, for each strand individually

Input: Library normalized coverage profiles e^r_{non} and e^r_{enr} for all conditions K
  Minimal percentile Q_{min}, Thresholds step height T_h, step factor T_f and enrichment factor T_{EF}
  Allowed shifts per replicate Δ_r, and conditions Δ_k, Reduction values ρ_h, ρ_f
  Minimal replicate support $R_{\min}$, Collapse window size W
  for all replicates r **do**
    $\mathcal{T}_r \leftarrow$ DETECTTSSPERREPLICATE$(e^r_{enr}, Q_{min}, T_h, T_f, W)$
  end for

  ▷ Cross-replicate grouping and threshold relaxation
  ▷ *If TSS have a position difference of at most Δ_r across replicates of a condition, account as equal*
  $\mathcal{T}_k \leftarrow$ GROUPWITHINCONDITIONS$(\mathcal{T}_r, \Delta_r)$
  for all clusters $\mathcal{T}_k$ with position j **do**
    for all replicates r not represented in $\mathcal{T}_k$ **do**
      $T'_f \leftarrow \max(1,\, T_f - \rho_f)$, $T'_h \leftarrow \max(0,\, T_h - \rho_h)$
      $T'^{\text{abs}}_h \leftarrow T'_h \cdot Q_{min}$
      for all $x \in [j - \Delta_r,\, j + \Delta_r]$ **do**
        $\Delta h \leftarrow e^r_{enr}(x) - e^r_{enr}(x-1)$, $F \leftarrow e^r_{enr}(x)/e^r_{enr}(x-1)$
        if $\Delta h \geq T'^{\text{abs}}_h$ **and** $F \geq T'_f$ **then**
          $\mathcal{T}_k \leftarrow \{\mathcal{K}_k, x\}$
          break
        end if
      end for
    end for
  end for
  ▷ *A TSS needs to occur at least in R_{min} replicated to be accounted*
  $\mathcal{T}^{detected}_k \leftarrow$ FILTERFORMINIMALOCCURRENCE$(\mathcal{T}_k, R_{min})$

  ▷ Enrichment factor evaluation
  $\mathcal{T}^{enriched}_k \leftarrow \emptyset$
  for all TSS in $\mathcal{T}^{detected}_k$ with position j **do**
    $EF \leftarrow$GETENRICHMENTFACTORACROSSREPLICATES$(\mathcal{T}_k(j), e^r_{k,non}, e^r_{k,enr})$
    if $EF \geq EF_{\text{thr}}$ **then**
      $\mathcal{T}^{enriched}_k \leftarrow \{\mathcal{T}^{enriched}_k, j\}$
    end if
  end for

  ▷ Cross-condition merging
  ▷ *If TSS have a position difference of at most Δ_k across conditions, account as equal*
  $\mathcal{T}^{enriched,detected} \leftarrow$GROUPACROSSCONDITIONS$(T^{enriched}_k, T^{detected}_k, \Delta_k)$
  return $\mathcal{T}^{enriched,detected}$
