# Supplementary material for: TSSpredator-Web: A web-application for transcription start site prediction and exploration
Source: PLoS One. 2026 Mar 13;21(3):e0326299. doi: 10.1371/journal.pone.0326299 (PMC12987492; doi:10.1371/journal.pone.0326299)
Supplement: Attachment — Submitted filename: Response to Reviewers.pdf [file pone.0326299.s006.pdf]

Journal Requirements

1. Please ensure that your manuscript meets PLOS ONE's style requirements, including those for file naming. The PLOS ONE style templates can be found at

https://journals.plos.org/plosone/s/file?id=wjVg/PLOSOne_formatting_sample_main_body.pdf and

https://journals.plos.org/plosone/s/file?id=ba62/PLOSOne_formatting_sample_title_authors_affiliations.pdf

2. Please note that PLOS ONE has specific guidelines on code sharing for submissions in which author-generated code underpins the findings in the manuscript. In these cases, we expect all author-generated code to be made available without restrictions upon publication of the work. Please review our guidelines at https://journals.plos.org/plosone/s/materials-and-software-sharing#loc-sharing-code and ensure that your code is shared in a way that follows best practice and facilitates reproducibility and reuse.

Reply: Our code is provided as a Github repository, with a DOI provided via Zenodo.

3. Thank you for stating in your Funding Statement:
"MWP and KN are supported by infrastructural funding from the Cluster of Excellence EXC 2124 'Controlling Microbes to Fight Infections' [project ID 390838134] from the DFG (Deutsche Forschungsgemeinschaft, German Research Foundation).

We acknowledge support from the Open Access Publication Fund of the University of Tübingen."

Please provide an amended statement that declares *all* the funding or sources of support (whether external or internal to your organization) received during this study, as detailed online in our guide for authors at http://journals.plos.org/plosone/s/submit-now. Please also include the statement "There was no additional external funding received for this study." in your updated Funding Statement.
Please include your amended Funding Statement within your cover letter. We will change the online submission form on your behalf.

Reply: We have adapted the funding and added the expected statement.

5. We note that Figure 3 in your submission contain copyrighted image. All PLOS content is published under the Creative Commons Attribution License (CC BY 4.0), which means that the manuscript, images, and Supporting Information files will be freely available online, and any third party is permitted to access, download, copy, distribute, and use these materials in any way, even commercially, with proper attribution. For more information, see our copyright guidelines: http://journals.plos.org/plosone/s/licenses-and-copyright.

We require you to either (1) present written permission from the copyright holder to publish these figures specifically under the CC BY 4.0 license, or (2) remove the figures from your submission:
a. You may seek permission from the original copyright holder of Figure 3 to publish the content specifically under the CC BY 4.0 license.
We recommend that you contact the original copyright holder with the Content Permission Form (http://journals.plos.org/plosone/s/file?id=7c09/content-permission-form.pdf) and the following text:
“I request permission for the open-access journal PLOS ONE to publish XXX under the Creative Commons Attribution License (CCAL) CC BY 4.0 (http://creativecommons.org/licenses/by/4.0/). Please be aware that this license allows unrestricted use and distribution, even commercially, by third parties. Please reply and provide explicit written permission to publish XXX under a CC BY license and complete the attached form.”
Please upload the completed Content Permission Form or other proof of granted permissions as an "Other" file with your submission.
In the figure caption of the copyrighted figure, please include the following text: “Reprinted from [ref] under a CC BY license, with permission from [name of publisher], original copyright [original copyright year].”

Reply: We are unsure to what extent this figure might contain copyrighted material, as we, ourselves, created this figure. It lists the requirements of TSSpredatorWeb and hence are unsure which other article should have published such a figure.

6. We are unable to open your Supporting Information file S1_DragDrop.eps and S2_RNAstructure.eps. Please kindly revise as necessary and re-upload.

Reply: we apologize for the inconvenience of using the postscript file format. We have now uploaded and thus provide all figures as PDFs.

8. Please review your reference list to ensure that it is complete and correct. If you have cited papers that have been retracted, please include the rationale for doing so in the manuscript text, or remove these references and replace them with relevant current references. Any changes to the reference list should be mentioned in the rebuttal letter that accompanies your revised manuscript. If you need to cite a retracted article, indicate the article’s retracted status in the References list and also include a citation and full reference for the retraction notice.

Reply: We have revised the reference list and completed some missing DOIs. We also are not citing any retracted papers.

Additional Editor Comments:

All three reviewers recommended only minor revisions. Congratulations! Please revised the manuscript accordingly to their comments.

Reply: Thank you, we highly appreciate that.

---

5. Review Comments to the Author

Reviewer #1:

TSSpredator is a useful tool for TSS mapping from prokaryotic RNA-seq data and this webportal is user-friendly in its design with a set of presets to alter sensitivity/specificity of the search. The replicate handling seems reasonable, and the user is provided with a range of files and visualization possibilities that make this webportal certainly valuable. The manuscript does a great job explaining all the options.

I cannot comment much on the building of the website as this is beyond my expertise.

There is one important point that remains unclear to me. The “coverage tracks” used by TSSpredator as input appear to be coverage tracks for the entire read length as judged from the figures 1, 4, 7 and 8. Limiting the coverage calculation to the 5’-ends of the reads that actually represent the TSS signal makes more sense and this option is included in the READemption software that the authors recommend for input file generation (“-b first_base_only”) as well as other widely used software like bedtools genomcov or deeptools . I know this rather refers to TSSpredator itself than the webportal described here. But, as a minimum, it should be properly defined in the text whether 5’-end coverage or read coverage (or fragment coverage for paired-end data

Reply: We thank the reviewer for pointing this out. Indeed, READemption offers both options for the computation of the coverages. To the best of our knowledge, the parameter for the “first base only” has not been used in other scientific publications when analysing TSS enrichment data, in particular when using TSSpredator on the basis of the READemption software. So our knowledge so far TSSpredator has is mainly used with coverage profiles accounting for the full read coverage (the default option in READemption). We added this information now in the manuscript (line 98). However, we point out, and have added this also in the manuscript (lines: 477-481), since TSSpredator does not conduct the step of coverage profile computation itself, the user is free to upload coverage profiles which limits the coverage calculation to the 5’ends of the reads. Nevertheless, we also would like to mention that our internal tests revealed, when comparing both approaches, that such coverage profiles might be more noisy, and lead to many more TSS calls, at least when using the default parameters of TSSpredator. Users therefore would need to choose more specific parameters, which however is not a problem in our program. We have also added this aspect in the discussion of our manuscript (lines:481-484).

Besides this point, I have only very minor comments.

Page 2, line 18 “most genome annotations only provide translation start sites” , maybe more precise to say “predicted translation start sites”, generally they are not experimentally verified.

Reply: Indeed, thank you, we have adapted the wording (in the introduction line 18).

Page 3, line 90 “pre-normalized” This is somewhat ambigous, raw coverage might be a better term here?

Reply: We thank the reviewer for pointing this out. Indeed, the term “raw coverage” is not correct in this case. The raw coverage profile is one of the outputs that READemption provides after coverage computation. However, we use one of the other two coverage files that are normalized: either by the total number of aligned reads and multiplied by the lowest number of aligned reads of all considered libraries (coverage-tnoar_min_normalized), or by the total number of aligned reads and multiplied by one million (coverage-tnoar_mil_normalized). We consider this pre-normalized as in TSSpredator another normalization step happens. However, we understand that this term might not be clear enough and have therefore adapted the wording in the manuscript (lines 103-107).

Page 8, line 286 Novobiocin is a DNA gyrase inhibitor, thus will affect more processes than “DNA synthesis”

Reply: We thank the reviewer for pointing this out, stating that our statement was too general in this regard. Indeed, Novobiocin inhibits the GyrB subunit of the DNA gyrase and therefore affects - among other things processes - the DNA replication. We have adapted the statement for this antibiotic and also expanded on the effects of the other two antibiotics for a clearer differentiation of these 3 antibiotics (lines: 315-317).

Page 9, line 324 “subset of TSS: those enriched only in the presence of an antibiotic” In the absensce of any statistical testing whether there is actually a significant difference here, I would rather say “passing the TSS calling threshold”.

Reply: We agree with the reviewer that the word “enriched” could be misleading and be interpreted as the result of a statistical analysis. Hence, we have adapted the statement to state that these 43 TSS were identified based on our TSS calling strategy (lines 352-355).

Reviewer #2:

This manuscript is well articulated and provides clear guidance for any usage of this tool.

One potential concern is that the algorithm underlying TSSPredator has been previously published, however the authors do not claim novelty with regard to this. In fact, the tool has been available for some time in a command-line interface.

Rather what the manuscript offers is increased usability of this tool through a streamlined process and integration with data visualization resources, which was not previously available. Indeed the authors outline 5 goals for the development of this GUI and clearly address how they accomplished each one.

Thus I think the manuscript potentially represents a valuable contribution to the community. Indeed the authors even informed their design of the tool based on common approaches used by other studies for data visualization, which is a thoughtful approach. However as the usability of the GUI is central to this project's impact it is important to note that the GUI is slow. This is not necessarily a problem for file upload and analysis as these are expected to take time, however when I was trying to work with the output the website kept crashing. This is a concerning issue for usability of this tool as one of its major assets is data visualization.

As the user can already download a set of results, it is worth considering also producing scripts for figure generation to support data visualization that could be easily run locally such as in R Studio (see http://revigo.irb.hr/ for an example of this style of implementation). This could be a workaround of website bandwidth issues if other options are not possible.

Reply: We thank the reviewer, in particular also for thoroughly testing the platform. Indeed, unfortunately we also have noticed this problem while exploring the data within the genome explorer outside of university networks, where the upload bandwidth is usually significantly lower than the download bandwidth. This issue only appears when using the genome visualization, which makes use of the Gosling library, therefore, also unfortunately, there is not much margin for caching or optimization. However, we have reduced the amount of API calls for specific tracks and have also made the read coverage visualization optional, as this visualization requires the highest amount of resources. With this we hope that the usability is much enhanced. In addition, we would like to emphasize that TSSpredator is also available via Docker images that can be run locally and therefore there is no dependency on the internet connection and the application runs faster and more smoothly. We added that statement in the manuscript (lines 226-229)

Comments:
lines 17-19 consider addressing why standard RNA-seq does not produce comprehensive results for TSS prediction

Reply: Standard RNA-seq is insufficient for accurate TSS identification, since it cannot distinguish between primary and processed transcripts. When a substantial fraction of transcripts is processed, the resulting 5′-end signal appears as a ladder-like pattern, which makes the precise location of the true TSS more difficult and complicates reliable prediction. We have added this and thus expanded the text to explain this aspect in more detail in the manuscript (lines 18-25).

lines 36-39 You make the claim that TSSs are essential for comparative transcriptomics. However comparative transcriptomics is accomplished without this. Consider more precise wording of what you mean by comparative transcriptomics.

Reply: We thank the reviewer for this comment. Indeed, this wording was not ideal. We have adapted our statement for a more precise formulation on how TSS can be applied for comparative transcriptomics (lines 40-44).

Discussion - consider slightly polishing the wording here. While fine and readable it read less polished than the other sections of the manuscript.

Reply: We have polished the discussion to improve readability (hopefully).

Change file type (e.g., png, jpeg, tiff) for supplementary materials so it is easier to view and does not have to be separately rendered.

Reply: Indeed, and we apologize for the inconvenience of using postscript as a file format. We have adapted the figures to a more compatible format (PNG & PDF).

Reviewer #3:

This manuscript presents the development of a user-friendly frontend for TSSPredator, a tool that identifies transcription starting sites from RNA sequencing data, called TSSPredator-Web. In addition to simplifying the prediction process for less technical researchers, TSSpredator-Web also provides an interface to explore the results dynamically. Finally, the authors demonstrate a use case of the tool to identify both known and potentially novel TSSs associated with antibiotic exposure.

This manuscript clearly describes the current state of the field and identifies the gap that their software is filling. The descriptions of the software and the TSSpredator algorithm are very clear. The use case showcases the capabilities of the software well and demonstrates clearly how a researcher could use it.

Reply: We thank the reviewer for this positive comment.

However, the use case could be expanded a bit to compare TSSpredator with the other mentioned TSS identification approaches. Overall I would recommend this manuscript be accepted with some minor revisions.

Though the authors describe the TSSpredator approach clearly in section 2.1, since it has not been formally published previously, they may consider making this work that publication and formalizing the algorithm, possibly using pseudo-code or taking a more mathematical approach.

Reply: We thank the reviewer for their suggestion. We have now expanded the explanation of the algorithm and have also included a pseudocode of the key steps (see Suppl. Algorithms 1-3).

Additionally, Figure 1 could be slightly elaborated to include where certain parameter choices are implicated. In fact, the authors might consider integrating Figures 1 and 2 to provide a more complete view of the workflow.

Reply: We thank the reviewer for this suggestion. Since both Figures 1 and 2 do not really address the question of how parameter choices affect the TSS calling, we have now added

two new Supplementary Figures, in which this aspect is explicitly documented and illustrated (see new Supplementary Figures 1 and 2).

The authors provide a use case of the tool in section 3 to demonstrate its capabilities over existing approaches. Near the end (line 355), the authors propose the potential identification of “previously unannotated transcriptional units.” However, it’s unclear if these are due to the new dataset or if they are unique to the TSSpredator algorithm

Reply: We thank the reviewer for pointing out this unclear statement. Here, we refer to transcriptional units that start at orphan TSS, meaning that no gene has been annotated by gene annotation pipelines downstream of this position. We do not state that this is unique to TSSpredator, as we have not compared other TSS calling approaches, but this is unique to methods that incorporate transcriptomic data. What is unique to TSSpredatorWeb, is that users are able to explore these regions even further, for example, by analyzing the sequence of promoter regions as well as the expression levels close to the TSS.

A comparison with the other aforementioned approaches would be useful. Overall, a summary of the differences between TSSpredator and the other approaches could put into context how the results from this approach differ.

Reply: Thank you. Indeed, a clearer differentiation between the TSSpredator approach and other approaches was missing from the manuscript. We have expanded our introduction to include the differences between the mentioned approaches and the underlying algorithm behind TSSpredator (lines 47-63).

The authors make clear that TSSpredator-Web is primarily for data exploration, and statistical evaluations of TSS identification are not elaborated on. However, potential researchers may be interested in demonstrating whether the existence of a particular TSS is statistically different between two or more experimental conditions. Though the tool may not be able to achieve this, it might be worth clarifying whether it would be possible to do this using the underlying data behind TSS identification. If not, it may be worth adding to the discussion of limitations.

Reply: Yes, indeed the main motivation for our manuscript was to provide a user-friendly tool for the computation of TSS based on TSSpredator and the subsequent exploration of the data and the results. However, your comment motivated us to investigate some effort into an additional aspect that so far has not been addressed with our tool, that is the possibility to statistically compare TSS between different conditions. We have not integrated this into the tool itself, however we provide scripts on our GitHub repository.

Our statistical approach uses the Kolmogorov-Smirnov test by comparing the coverage distributions around the called TSS between the conditions. For this, we conduct pairwise KS-tests between all replicates of the conditions to be compared and compute a common p-value using the Cauchy combination test. Finally, all the combined p-values of all tested TSS are subject to a Bonferroni p-value correction. This approach is also now described in the discussion of our manuscript (lines 463-472).
